# An adenovirus-based multiantigen vaccine protects against tuberculosis by coordinating adaptive immunity and curbing immunosuppressive mechanisms

**DOI:** 10.1128/spectrum.00907-26

**Published:** 2026-08-28

**Authors:** Liqun Wei, Peng Wang, Ying Li, Xuejun Li, Xiaoling Shen, Furong He, Yingying Guo, Man Xing, Dongming Zhou

**Affiliations:** 1 Department of Pathogen Biology, School of Basic Medical Sciences, Tianjin Medical University 12610, Tianjin, China; 2 Shanghai Public Health Clinical Center, Fudan University, Shanghai, China; ICON plc, London, United Kingdom

**Keywords:** *M. tuberculosis*, chimpanzee adenovirus vector, multi-antigen, autophagy, antigen presentation, T-cell

## Abstract

Given that tuberculosis (TB) remains the leading cause of death from a single infectious agent, and that vaccination is the most effective control strategy, we systematically compared several vaccine construction strategies using a chimpanzee adenovirus vector platform. This led to the identification of a multi-antigen vaccine candidate, Ad-8^CP^R2, as the most immunogenic construct. This candidate incorporates *M. tuberculosis* Ag85B, RpiB, and Rv2628 fused with the autophagy-inducing peptide C5 and Pan DR-binding epitope (PADRE) peptide. The C5 peptide in Ad-8^CP^R2 vaccine enhanced the autophagic activity of antigen-presenting cells, thereby enhancing antigen presentation via the MHCI pathway and eliciting broad, multidimensional T-cell responses. Notably, Ad-8^CP^R2 promoted the enrichment of tissue-resident memory T cells in the lungs, establishing local mucosal immunity that may restrict *M. tuberculosis* dissemination, while also generating antibodies capable of preventing *M. tuberculosis* infection. Furthermore, Ad-8^CP^R2 limited the accumulation of myeloid-derived suppressor cells and alleviated T-cell exhaustion, thereby preserving adaptive immune function during chronic infection. Ultimately, we identified Th1 and Th17 responses, alongside functionally relevant antibodies, as key correlates of vaccine-induced protection.

**IMPORTANCE:**

Current efforts in developing novel tuberculosis vaccines are to enhance the protective efficacy of vaccine candidates, with research primarily centered on rational vaccine design strategies for the generation of multivalent antigen constructs. In this context, we propose an approach involving the selection of *M. tuberculosis* antigenic proteins with strong immunogenicity and functional properties, which are then fused with peptide segments capable of augmenting antigen-specific immune responses. Delivery of these constructs via viral vector platforms induces robust and durable adaptive immunity. Such a strategy may further improve vaccine-mediated protection and could provide a theoretical foundation for developing next-generation tuberculosis vaccine candidates.

## INTRODUCTION

*Bacillus Calmette-Guérin* (BCG), the only licensed preventive vaccine for tuberculosis (TB), has been widely administered since its development in 1921 (1). Unfortunately, TB remains the leading cause of death from a single pathogen infection globally. In recent years, the global burden of TB has continued to rise, with approximately 8 million new cases reported annually (2), posing a persistent and major challenge to global health (3). The increasing incidence of drug-resistant TB, coupled with poor treatment adherence among patients receiving long-term therapy, has further undermined efforts to control TB transmission through clinical intervention alone (4). Vaccination represents the most effective means of preventing TB transmission, and the development of novel TB vaccines utilizing advanced vaccine platforms is essential to ending the TB epidemic.

Typically, the protective efficacy of BCG lasts only 7 to 10 years in infants and children (5), and its protection against pulmonary TB varies widely among individuals, ranging from 0% to 80% (6). Furthermore, in adults—the primary population at risk for active TB—the BCG vaccine fails to provide effective protection against pulmonary TB. This is also a significant factor contributing to the transmission of TB within the population. To date, significant progress has been made in TB vaccine research across multiple technical routes, with several vaccines based on recombinant BCG, protein subunits, and mRNA vectors entering clinical trials. The recombinant BCG vaccine VPM1002, developed by the Max Planck Institute in Germany, has been evaluated in neonates and adults in India and African nations, demonstrating improved safety and potential applicability even in immunocompromised individuals (7, 8). However, a multicenter trial in South Africa demonstrated that VPM1002 exhibits lower immunogenicity in newborns compared with the BCG vaccine (9). The recombinant protein subunit vaccine M72/AS01E, led by GlaxoSmithKline, entered Phase III clinical trials in Africa and Southeast Asia in 2023. Phase IIb clinical trials show that its protective efficacy against pulmonary TB recurrence in latently infected individuals is approximately 49.7% (10), falling short of the WHO’s target of >50% efficacy (11). These findings highlight the need to expand the repertoire of *M. tuberculosis* antigens for vaccine development, and to improve vaccine efficacy through innovative design, in alignment with the WHO goal of TB elimination (12–14).

In this study, we selected three *M. tuberculosis* antigens and fused two peptides. Ag85B is an early secreted antigen involved in cell wall integrity and facilitates *M. tuberculosis* adhesion to macrophages via fibronectin binding (15). Knockdown or overexpression of the Ag85B gene resulted in decreased or increased ability of BCG strains to infect macrophages, respectively, compared with wild-type BCG (16). RpiB, a metabolic regulatory protein involved in cell wall synthesis, exhibits strong immunogenicity. Previous studies have shown that the lipoarabinomannan (LAM) complex on the outer cell wall of *M. tuberculosis* inhibits phagosome-lysosome fusion in macrophages, thereby promoting intracellular bacterial survival (17); it also suppresses IL-12 production, leading to negative regulation of Th1-type immune responses (18). RpiB, a key enzyme in the DPA biosynthesis pathway, directly participates in LAM synthesis (19), suggesting that this antigenic protein may hold potential as a target for counteracting immune evasion by *M. tuberculosis*. Rv2628 is a latency-expressed protein regulated by the dormancy survival regulator under hypoxic conditions and can induce both Th1 and Th17 immune responses, thus being widely used in vaccine development (20). Pan DR-binding epitope (PADRE), as a synthetic universal T helper epitope, is widely used in vaccine studies to enhance CD4^+^ T-cell responses and antigen-specific antibody titers (21). The autophagy-inducing peptide C5, derived from amino acids 35–75 of the early secreted antigen CFP10, can activate the TLR2 pathway and upregulate cellular autophagy activity (22). Tandem fusion of C5 with *M. tuberculosis* antigens has been shown to enhance antigen-specific T-cell immune responses (23).

The chimpanzee adenovirus ChAd68 represents a promising vaccine vector due to its high transduction efficiency, broad cell tropism, robust gene expression, genetic stability, and low seroprevalence in humans (24). Based on this platform, we constructed novel TB vaccine candidates by expressing the *M. tuberculosis* antigens Ag85B, RpiB, and Rv2628. These antigens were genetically fused in tandem with the PADRE and C5 as molecular adjuvants. Here, we systematically evaluated the immunogenicity and protective efficacy of TB vaccine candidates generated through different construction strategies. We identified an optimal vaccine candidate, Ad-8^CP^R2, which elicits broad, multidimensional T-cell responses. Comprehensive analysis of adaptive immune responses and protection against infection further revealed key determinants of vaccine immunogenic potency.

## RESULT

### Construction and *in vitro* expression of multi-antigen TB vaccines

**Fig 1:**
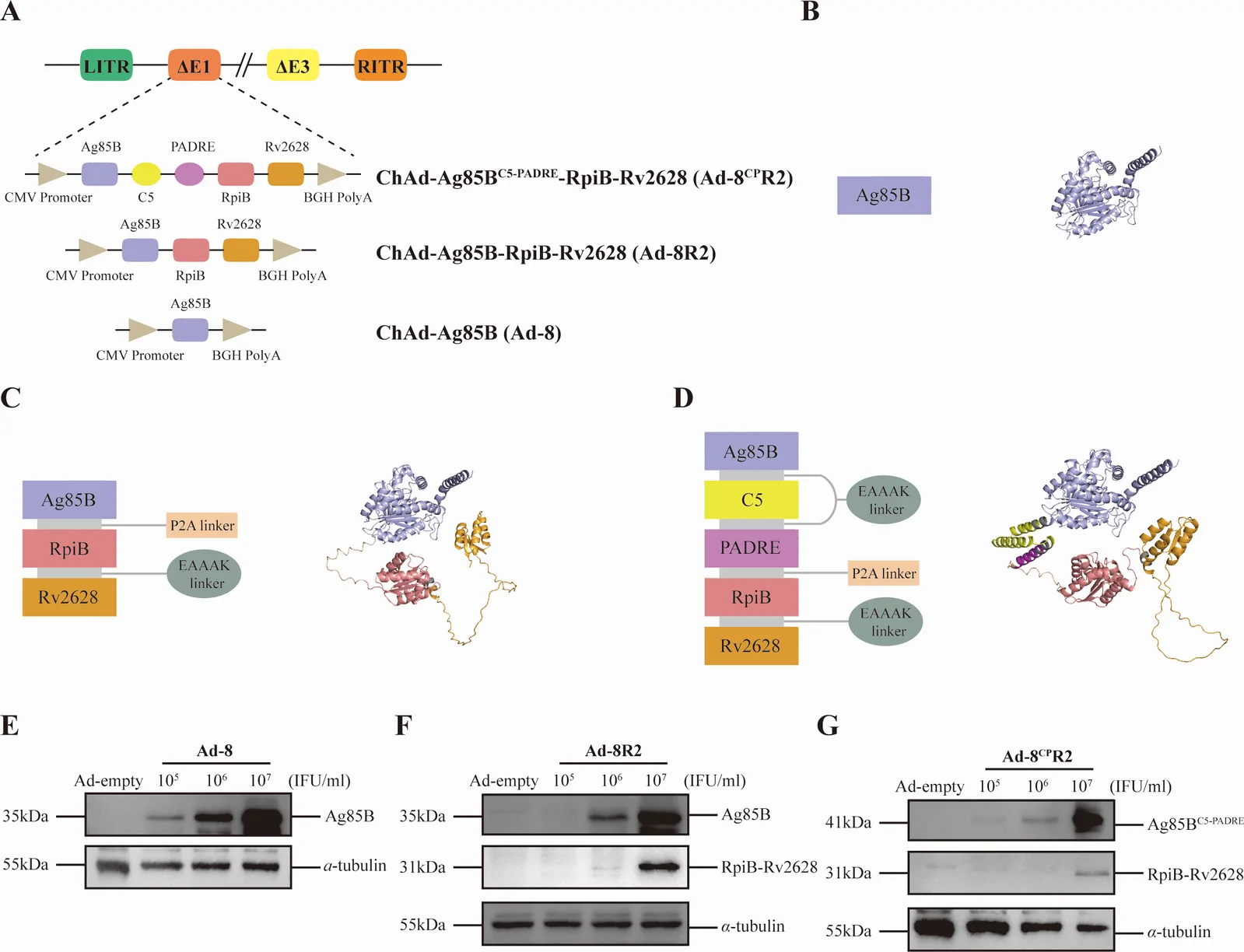
Construction and *in vitro* expression of multi-antigen TB vaccines. (**A**) Schematic of vaccine candidates’ construction. Ag85B, RpiB, Rv2628 proteins from *M. tuberculosis*, and C5, PADRE peptides were inserted into E1 deletion region of ChAd68 vector. (**B–D**) Predicted structures of the tandem antigens in each vaccine candidate. (**E–G**) Expression analysis of the indicated antigens from each vaccine candidate. HEK-293 cells infected with 10^5^, 10^6^, or 10^7^ IFU of each recombinant adenovirus were collected 24 h post-infection. Antigen expression was assessed by Western blot analysis. Cells infected with the Ad-empty vector (10^7^ IFU) served as the negative control. Representative blots from three independent experiments are shown.

We constructed three vaccine candidates based on the chimpanzee adenovirus vector ChAd68: a single-antigen vaccine, ChAd68-Ag85B (Ad-8); a multi-antigen vaccine, ChAd68-Ag85B-RpiB-Rv2628 (Ad-8R2); and a multi-antigen vaccine incorporating the C5 peptide and PADRE fused in tandem with the Ag85B antigens: ChAd68-Ag85B^C5-PADRE^RpiB-Rv2628 (Ad-8^CP^R2) (Fig. 1A). The spatial structures of antigenic proteins encoded by the three vaccines were predicted using AlphaFold3, illustrating the linkage between antigens and the molecular adjuvants (Fig. 1B through D). To confirm protein expression, HEK-293 cells were infected with each vaccine candidate. Western blot analysis revealed that all three vaccine candidates expressed the corresponding target proteins, with protein expression levels exhibiting a dose-dependent manner (Fig. 1E through G).

### ChAd TB vaccines induce durable and robust antibody responses

**Fig 2:**
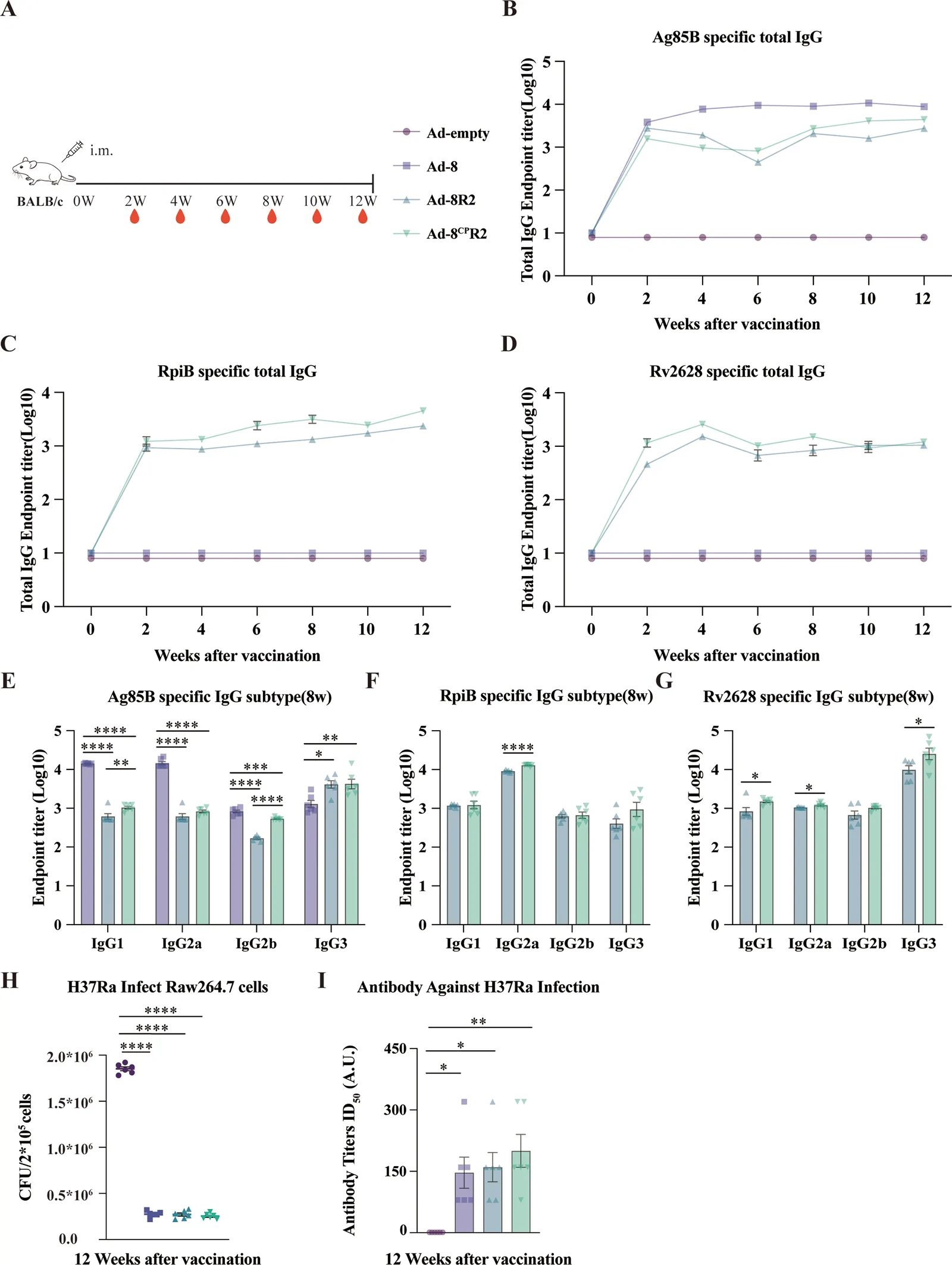
ChAd TB vaccines induce durable and robust antibody responses. (**A**) Scheme of experiments. Mice (*n* = 6) were immunized administered via intramuscularly (i.m.) injection with 1 × 10^8^ IFU of each vaccine candidate, Ad-empty was used as control. Serum samples were collected periodically at the indicated time points. (**B–D**) The kinetics of binding antibody response to Ag85B (**B**), RpiB (**C**), Rv2628 (**D**). Data are presented as endpoint titers. (**E–G**) IgG subclasses analysis. Serum collected 8 weeks post-vaccination was analyzed for antigen-specific IgG1, IgG2a, IgG2b, and IgG3 titers against Ag85B (**E**), RpiB (**F**), Rv2628 (**G**). (**H**) RAW 264.7 cells were infected with H37Ra incubating with serum (12 weeks post-vaccination) 5 h (MOI 1:10) for 6 h at 37°C and intracellular bacterial load determined. (**I**) Serum anti-H37Ra-eGFP infected antibody titers at 12 weeks post-vaccination among three vaccine candidates (100 CFU/10^6^ cells). Statistical analysis was conducted using unpaired Student’s *t*-test (for two-group analyses) (**F and G**) or one-way ANOVA with Tukey correction (**E, H, and I**) and represented as mean ± SEM. *P ≤ 0.05, **P ≤ 0.01, ***P ≤ 0.001, and ****P ≤ 0.0001. All data were pooled from two independent experiments.

BALB/c mice were immunized intramuscularly with a single dose of each vaccine candidate or an empty ChAd68 vector control (Ad-empty). Serum was collected biweekly for 12 weeks to monitor the kinetics of antigen-specific antibody response (Fig. 2A). Compared with the control group, all three candidates induced high levels of antigen-specific binding antibody responses that remained stable through the 12-week endpoint without significant decline (Fig. 2B through D). Ad-8 induced significantly higher levels of Ag85B-specific antibody compared with the multi-antigen groups Ad-8R2 and Ad-8^CP^R2. Between the multi-antigen groups, Ad-8^CP^R2 induced higher antigen-specific binding antibody titers than Ad-8R2. To further characterize the binding antibody response, we measured the titers of antigen-specific IgG subclasses (IgG1, IgG2a, IgG2b, IgG3) by ELISA in serum collected at 8 weeks post-immunization. All vaccines induced robust titers of these subclasses (Fig. 2E through G), which are known to contribute differentially to anti-mycobacterial immunity.

Since *M. tuberculosis* initially infects alveolar macrophages after entering the lung (25), we assessed the functional capacity of vaccine-induced antibodies to reduce bacterial infectivity. Serum collected 12 weeks post-immunization was diluted tenfold, pre-incubated with the attenuated *M. tuberculosis* strain H37Ra for 5 h, and then co-cultured with Raw264.7 macrophages. Compared with the control serum, serum from all vaccinated groups effectively reduced the number of H37Ra colony-forming units (CFUs) in macrophages (Fig. 2H). To further compare functional potency across vaccine candidates, GFP-expressing H37Ra was pre-incubated with serially diluted serum prior to macrophage infection, and the infection rate was quantified by fluorescence microscopy. Serum from Ad-8^CP^R2-immunized mice demonstrated the strongest capacity to inhibit TB infection (Fig. 2I).

Taken together, all vaccine candidates induced durable and potent antigen-specific antibody responses, and the antibodies induced by Ad-8^CP^R2 showed the strongest ability to reduce M. tb infection.

### Multi-antigen TB vaccine induce stronger T-cell immune responses

**Fig 3:**
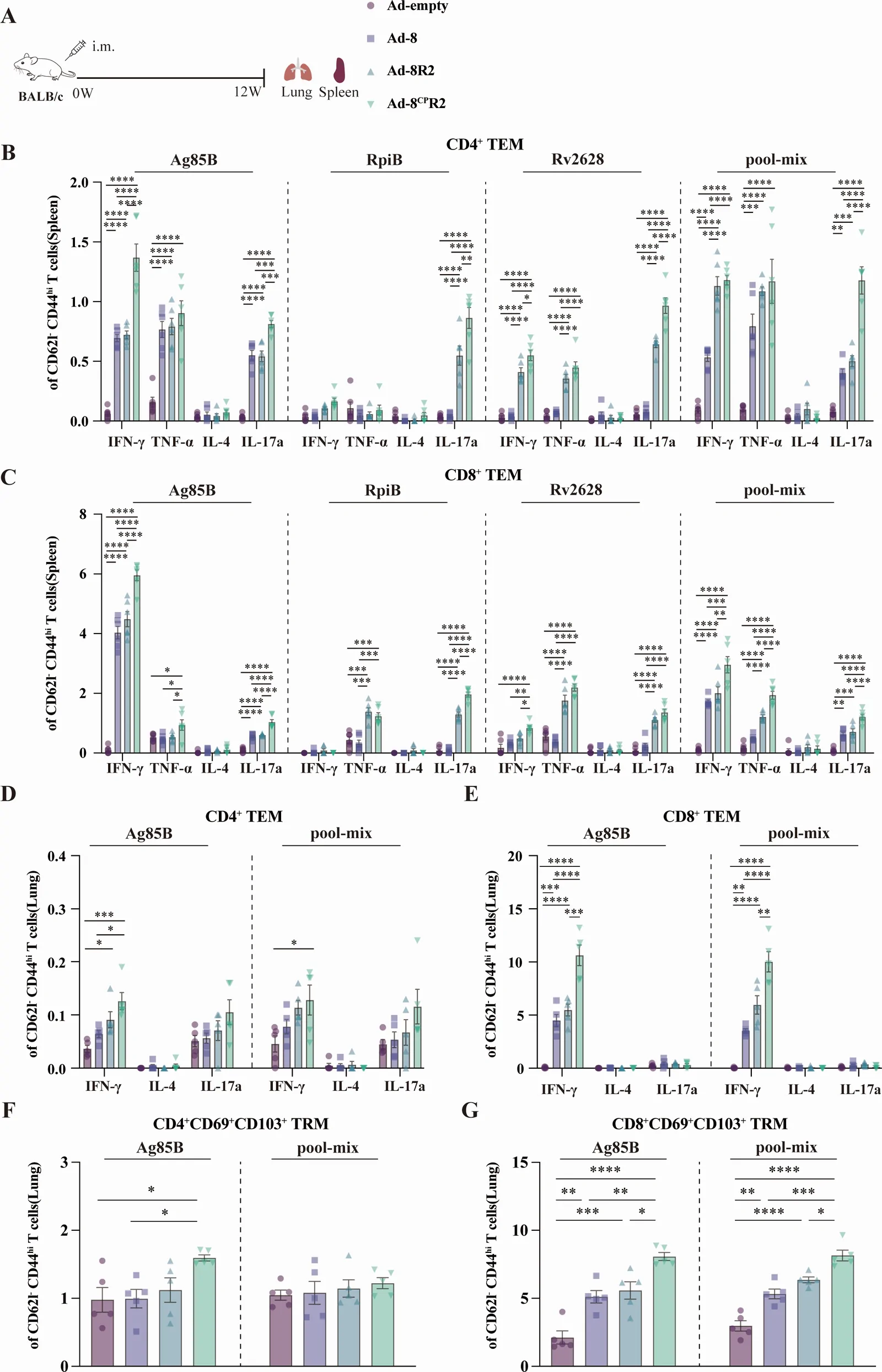
Multi-antigen TB vaccines induce stronger T-cell immune responses. (**A**) Immunization schedule. Mice were i.m. immunized with 1 × 10^8^ IFU of each vaccine candidate. Ad-empty was used as control (1 × 10^8^ IFU). (**B and C**) The frequencies of cytokine-producing, antigen-specific CD4^+^ (**B**) and CD8^+^ TEM cells (**C**) in the spleens. Splenocytes were isolated form BALB/c (*n* = 6) for analysis at 12 weeks post-immunization. Lymphocytes were stimulated with peptide pools specific for Ag85B, RpiB, Rv2628, or pool-mix. (**D and E**) The frequencies of cytokine-producing, Ag85B-specific and pool-mix-specific CD4^+^ (**D**) and CD8^+^ TEM cells (**E**) in the lungs of mice (*n* = 5) were quantified. (**F and G**) The frequencies of CD4^+^ (**F**) or CD8^+^ (**G**) TRM cells in the lungs of mice (*n* = 5) following stimulation with Ag85B or pool-mix were quantified. All data were pooled from two independent experiments and shown as means ± SEM. *P*-values were analyzed by one-way ANOVA with Tukey correction. *P < 0.05; **P < 0.01; ***P < 0.001; ****P < 0.0001.

To systematically evaluate long-term cellular immune memory, lymphocytes were isolated from the spleens and lungs of mice 12 weeks post-immunization (Fig. 3A). Cells were stimulated with peptide pools corresponding to Ag85B, RpiB, Rv2628, or a mixed pool of all three antigens (pool-mix). Effector memory T cells (TEM), central memory T cells (TCM), and TRM were analyzed to characterize vaccine-mediated memory T-cell responses. In the spleens, all three candidates elicited antigen-specific T-cell responses, with the strongest response against Ag85B. Antigen-specific T cells predominantly produced Th1-type cytokines (IFN-γ, TNF-α) and the Th17-type cytokine IL-17a. CD8^+^ T-cell responses were markedly more robust than CD4^+^ T-cell responses. Compared with the single-antigen vaccine, multi-antigen vaccine candidates induced stronger cellular immunity, the RpiB antigen induced IL-17a secretion by splenic CD4^+^ T cells, driving a Th17-type T-cell response, and stimulated CD8^+^ T cells to produce the intracellular cytokines TNF-α and IL-17a, mediating Th1 and Th17 responses; similar to Ag85B, Rv2628 also induced T cells to secrete IFN-γ and TNF-α, mediating multi-dimensional Th1-type immunity, as well as a predominantly IL-17a-driven Th17-type response, with the molecular adjuvant-containing vaccine Ad-8^CP^R2 eliciting the most potent T-cell responses (Fig. 3B and C). In contrast to TEM, splenic TCM levels remained at baseline without significant activation (Fig. S1B and C).

Lung lymphocytes stimulated with Ag85B or pool-mix showed a response pattern similar to that observed in splenocytes: vaccine-induced immunity was primarily mediated by CD8^+^ T cell activation, and Ad-8^CP^R2 again induced the strongest response (Fig. 3D and E). However, unlike in the spleens, TNF-α^+^ CD4^+^ T cells in the lungs were not significantly activated; Th1 responses were driven predominantly by IFN-γ^+^ CD4^+^ T cells. Furthermore, while IL-17a production was detected in CD4^+^ T cells, CD8^+^ T cells showed minimal IL-17a expression, indicating that Th17-type immune responses were mainly mediated by CD4^+^ T cells (Fig. 3D). To evaluate whether the vaccines could establish potent local immune protection at the primary infection site, TRM cells were identified by the CD69^+^CD103^+^ phenotype (26). Flow cytometric analysis showed that upon Ag85B or pool-mix stimulation, Ad-8^CP^R2 significantly activated CD4^+^ TRM enrichment under Ag85B stimulation (Fig. 3F) and induced a greater accumulation of CD8^+^ TRM cells in the lungs compared with other candidates (Fig. 3G).

In summary, during the memory phase, all vaccine candidates exerted memory T-cell immune responses mainly by the activation of IFN-γ^+^ CD8^+^ T cells mediating antigen-specific immune responses. Ad-8^CP^R2 broadened the diversity of memory T-cell immunity and demonstrated superior immunoprotective potential, notably, by enhancing the accumulation of CD8^+^ TRM cells in the lungs, which is a key feature for limiting pulmonary TB progression (27).

### Ad-8^CP^R2 enhances adaptive immunity by activating autophagy and promoting APC activation

**Fig 4:**
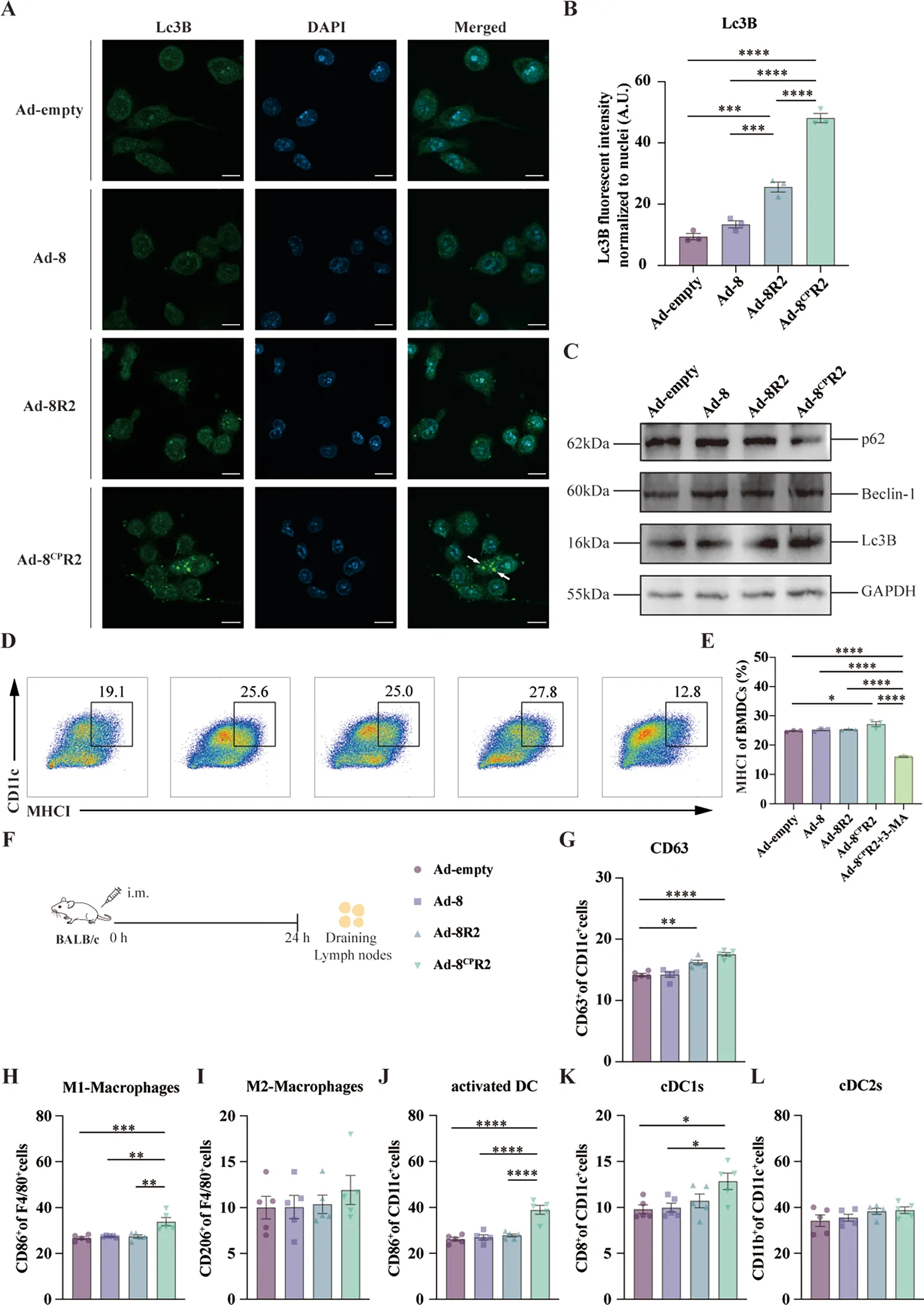
Ad-8^CP^R2 enhances adaptive immunity by activating autophagy and promoting APCs activation. (**A**) Autophagy induction in DC2.4 cells. DC2.4 cells were infected with each vaccine candidate or the Ad-empty control at MOI of 10. At 4 h post-infection, cells were fixed and stained with antibodies specific for autophagosome marker Lc3B (white arrow). Representative images from *n* = 3 independent experiments. (**B**) Quantification of LC3B fluorescence intensity. For quantification, nine non-overlapping fields of view were analyzed per condition from three biological replicates. (**C**) Expression analysis of autophagy-related proteins (p62, Beclin-1, Lc3B). BMDCs were infected with indicated vaccine candidates and collected for Western blotting analysis at 18 h post-infection. Representative blots from three independent experiments are shown. (**D**) Representative flow cytometry plots of live cells gated for CD11c^+^ and MHCI^+^ BMDCs. (**E**) Quantification of the proportion of MHCI^+^ BMDCs. Data from *n* = 3 independent experiments, each with three technical replicates. (**F**) Immunization schedule. Mice (*n* = 5 per group) were i.m. immunized with 1 × 10^8^ IFU of each vaccine candidate or the Ad-empty control. (**G–L**) Flow cytometry analysis of surface molecules on immune cells from draining lymph nodes. All data were pooled from two independent experiments and shown as mean ± SEM. Statistical analysis was performed using one-way ANOVA with Tukey’s post-hoc correction (**E–I**). *P ≤ 0.05, **P ≤ 0.01, ***P ≤ 0.001, and ****P ≤ 0.0001. Scale bars: 20 µm (**A**).

To investigate how the multi-antigen vaccine Ad-8^CP^R2 induces a more potent adaptive immune response, we investigated the functional contribution of the autophagy-inducing C5 peptide within the ChAd vaccine platform. DC2.4 cells were infected with the three vaccine candidates, and the expression level of the autophagy marker protein LC3B was assessed by fluorescence microscopy. At 4 h post-infection, DC2.4 cells infected with Ad-8^CP^R2 exhibited the most intense green fluorescence signal (Fig. 4A). Quantitative analysis confirmed that the fluorescence intensity in the Ad-8^CP^R2 group was significantly higher than in all other groups (Fig. 4B). Autophagy pathway activation was further evaluated in mouse bone marrow-derived dendritic cells (BMDCs) by WB. Compared with other vaccine candidates, infection with Ad-8^CP^R2 upregulated the expression levels of LC3B and Beclin-1 proteins, while downregulating p62 (Fig. 4C), indicating that Ad-8^CP^R2 enhances autophagy activity in antigen-presenting cells (APCs).

To determine whether increased autophagy influenced antigen presentation, we analyzed major histocompatibility complex (MHC) expression on BMDCs. Ad-8^CP^R2 infection elevated the proportion of MHCI^+^ BMDCs, an effect that was significantly reduced by treatment with the autophagy inhibitor 3-methyladenine (3-MA) (Fig. 4D and E). In contrast, the proportion of MHCII^+^ BMDCs remained unchanged (Fig. S2). These findings suggested that increased autophagic activity leads to elevated endogenous antigen presentation and cross-presentation efficiency.

To validate these findings *in vivo*, mice were immunized intramuscularly, and lymphocytes from draining lymph nodes were analyzed 24 hours later (Fig. 4F). Immunization with Ad-8^CP^R2, compared with other groups, increased the proportion of macrophages polarized towards the M1 macrophages, which is associated with antigen presentation (Fig. 4H and I), and enhanced dendritic cell activation accompanied by increased cytoplasmic vesicle generation (Fig. 4G through J). Consistent with *in vitro* results, the proportion of cDC1s, responsible for endogenous and cross-presentation, was elevated among DCs from Ad-8^CP^R2-immunized mice (Fig. 4K through L).

Collectively, these results indicate that Ad-8^CP^R2 enhances endogenous and cross-presentation efficiency by upregulating autophagy activity both *in vitro* and *in vivo*, thereby potentiating the antigen-specific adaptive immune response.

### TB vaccine candidates reduce bacterial burden and alleviate inflammatory responses

**Fig 5:**
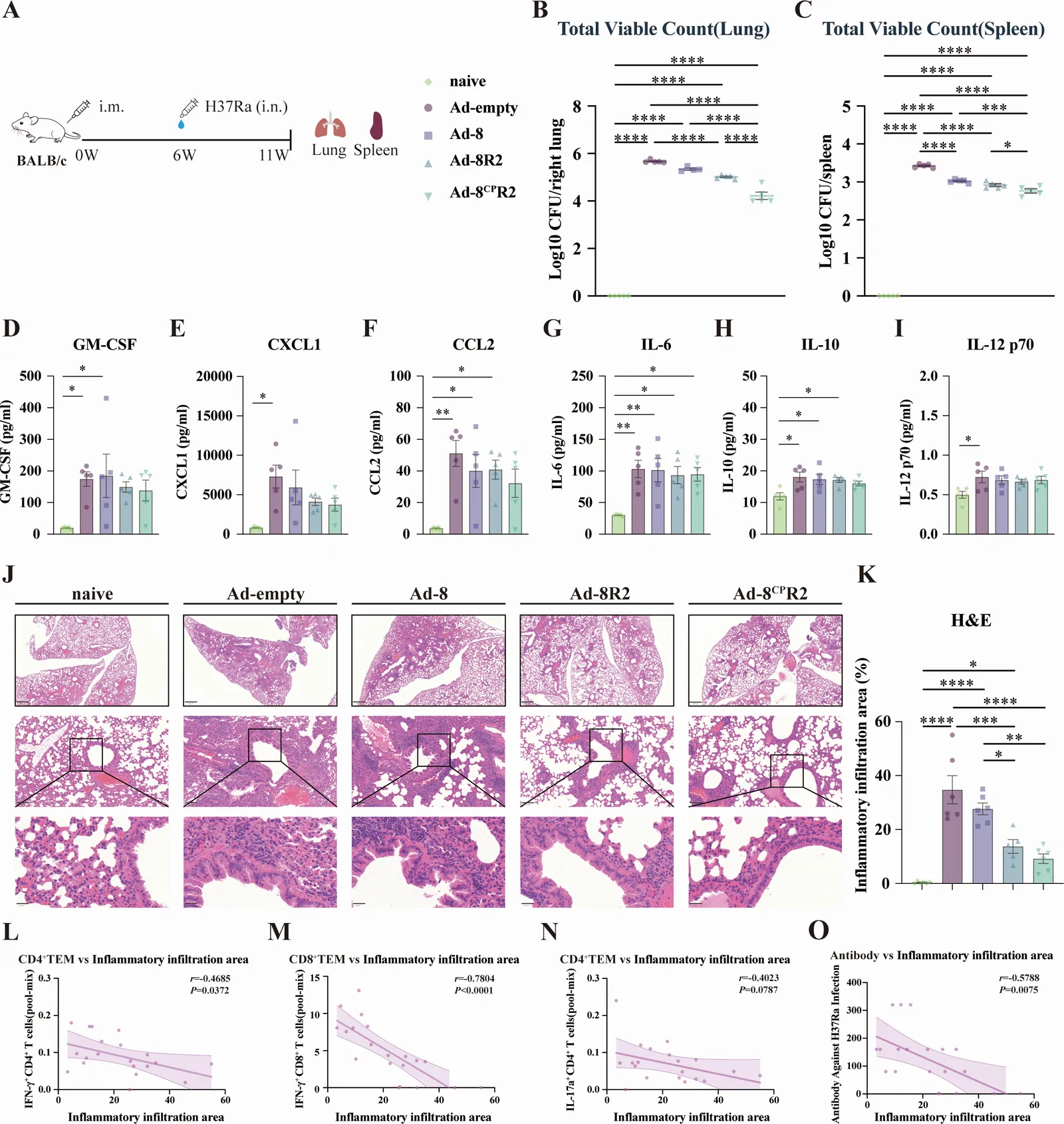
TB vaccine candidates reduce bacterial burden and alleviate inflammatory responses. (**A**) Scheme of vaccination and bacterial challenge. Mice (*n* = 5 per group) were i.m. immunized with 1 × 10^8^ IFU of each vaccine candidate or Ad-empty. The uninfected naive mice were used as negative controls. At 6 weeks post-vaccination, mice were intranasally (i.n.) challenged with 1 × 10^6^ CFU of H37Ra. At 5 weeks post-infection, mice were euthanized, and lung and spleen tissues were collected. (**B and C**) Bacterial burden in the lungs and spleens. The CFU of H37Ra in the lungs (**B**) and spleens (**C**) of infected mice at 6 weeks post-infection were assessed. (**D–I**) Levels of inflammatory cytokines in the lungs of infected mice at 6 weeks post-infection were measured by ELISA. (**J**) H&E staining for pathological examination. Representative magnified images are shown. (**K**) Quantification of inflammatory infiltration. The area of inflammatory infiltration in the lungs of BALB/c mice (*n* = 6 per group) was quantified using Fiji software. (**L–N**) Spearman correlation analysis between the frequencies of IFN-γ^+^ CD4^+^ TEM (**L**), IFN-γ^+^ CD8^+^ TEM (**M**), IL-17a^+^ CD4^+^ TEM (**N**), antibody against H37ra (**O**), and the inflammatory infiltration area. All data were pooled from two independent experiments and shown as mean ± SEM. Statistical analysis was conducted using one-way ANOVA followed by Tukey’s correction. *P ≤ 0.05, **P ≤ 0.01, ***P ≤ 0.001, and ****P ≤ 0.0001. Scale bars: top 20 µm; middle 100 µm; bottom 500 µm (**J**).

To evaluate the preventive efficacy of the vaccine candidates, BALB/c mice immunized six weeks earlier were challenged intranasally with 1 × 10^6^ CFU of *M. tuberculosis* strain H37Ra. Lungs and spleens were harvested 5 weeks post-challenge to quantify bacterial burden by colony plating (Fig. 5A). Compared with uninfected naive mice, H37Ra colonization was detected in all infected mice. Vaccination with the vaccine candidates significantly reduced the bacterial loads in the lungs and spleens, with the lowest colony counts observed in mice immunized with Ad-8^CP^R2 (Fig. 5B and C).

To evaluate pulmonary inflammation, supernatants from lung homogenates of infected mice were analyzed for cytokine levels by ELISA. Infected mice exhibited significantly elevated inflammatory cytokines relative to naive controls. Multi-antigen vaccines were more effective than the single-antigen vaccine (Ad-8) in reducing lung inflammation, with the Ad-8^CP^R2 group showing the lowest cytokine concentrations, indicating superior control of pulmonary inflammation (Fig. 5D through I). Histopathological analysis of lung sections by H&E staining revealed thickened bronchiolar walls and increased peribronchiolar inflammatory infiltration in infected mice. The Ad-empty control group showed extensive destruction of alveolar structure. Compared with the single-antigen vaccine group, lungs from multi-antigen vaccine groups displayed clearer alveolar structures and markedly reduced bronchiolar wall thickening (Fig. 5J). Quantitative analysis of inflammatory infiltration area confirmed the smallest area in the Ad-8^CP^R2 group (Fig. 5K).

Furthermore, we performed correlation analysis between vaccine-induced immune parameters—including antibodies capable of inhibiting H37Ra infection, pulmonary Th1 responses (frequencies of IFN-γ^+^ CD4 and CD8^+^ TEM cells), and Th17 responses (frequencies of IL-17a^+^ CD4^+^ TEM cells)—and the lung inflammatory infiltration post-infection. Results showed negative correlations, indicating that stronger antibodies, Th1, and Th17 cellular immunity correlates with reduced lung pathology (Fig. 5L through O). These findings suggest that functionally relevant antibodies and T cells secreting IFN-γ and IL-17 mitigate inflammatory damage and contribute to protective immunity against *M. tuberculosis*.

In conclusion, all three TB vaccine candidates activated pulmonary T cells to secrete IFN-γ and IL-17, mediating Th1 and Th17 responses that conferred immunoprotection and limited inflammatory infiltration. Ad-8^CP^R2 was most effective in preventing *M. tuberculosis* infection, suppressing pulmonary cytokine release, and attenuating pathological damage, demonstrating its superior anti-*M*. *tuberculosis* protective potential.

### TB vaccine candidates confer protective effects by regulating immune cell functions

**Fig 6:**
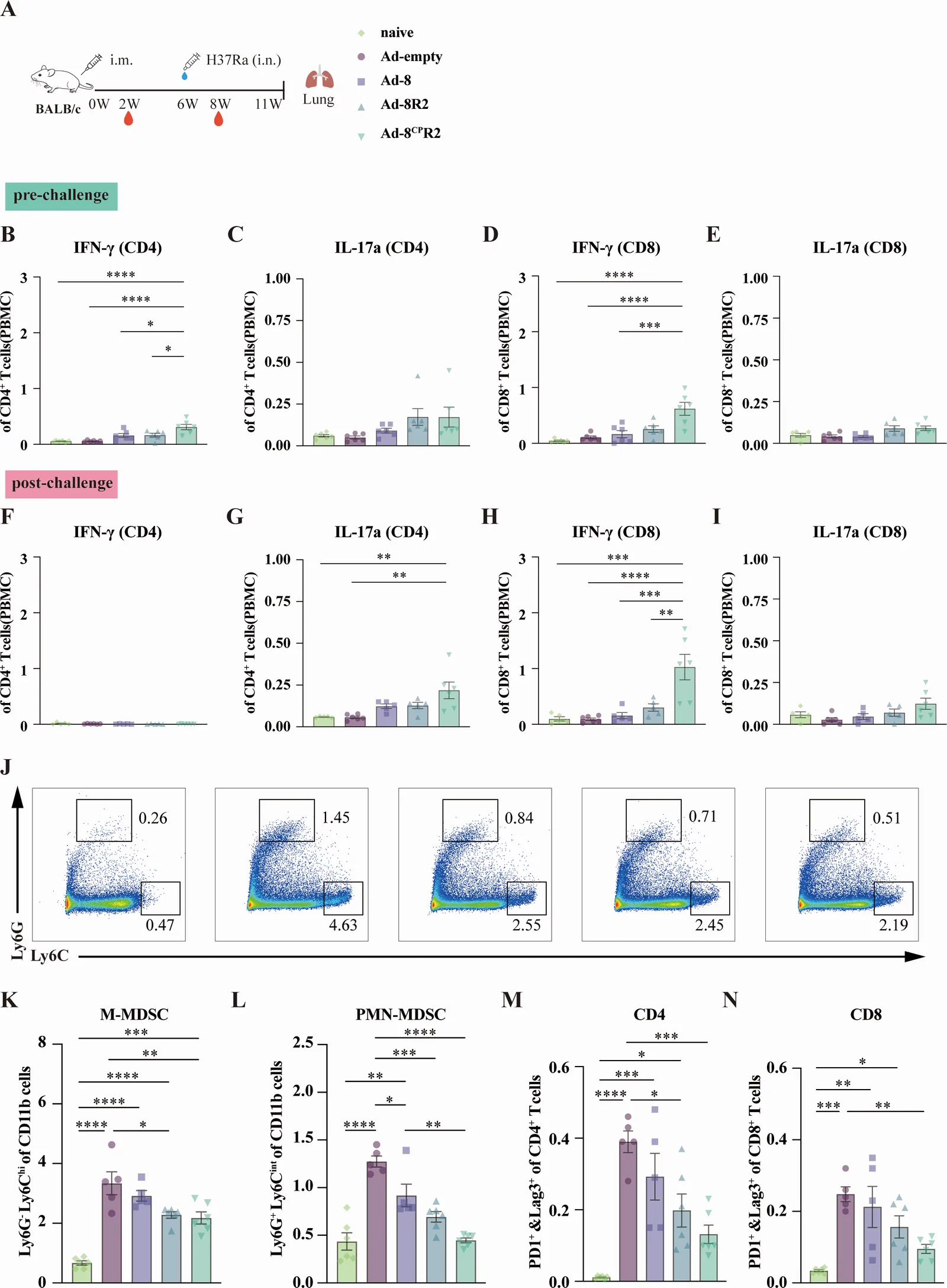
TB vaccine candidates confer protective effects by regulating immune cell function. (**A**) Scheme of vaccination and bacterial challenge. (**B–E**) T-cell responses post-immunization. PBMC were isolated form BALB/c (*n* = 6 per group) at 2 weeks post-immunization. Lymphocytes were stimulated with pool-mix. The frequencies of cytokine-producing, antigen-specific CD4^+^ (**B and C**) and CD8^+^ T cells (**D and E**) were quantified. (**F–I**) T-cell responses post-challenge. PBMC were isolated form BALB/c at 2 weeks post-challenge. Lymphocytes were stimulated with pool-mix. The frequencies of cytokine-producing, antigen-specific CD4^+^ (**F and G**) and CD8^+^ T cells (**H and I**) were quantified. (**J**) MDSC gating strategy. Representative flow cytometry plots of CD45^+^ lung cells from BALB/c mice (*n* = 5 per group), gated for M-MDSCs (Ly6G^-^Ly6C^hi^) and PMN-MDSCs (Ly6G^+^Ly6C^int^). (**K and L**) Quantification of M-MDSCs (**K**) and PMN-MDSCs (**L**) in the lungs. (**M and N**) T-cell exhaustion in the lungs. The frequencies of exhausted CD4^+^ T cells (**M**) and CD8^+^ T cells (**N**) in the lungs from BALB/c mice (*n* = 5) were quantified. All data were pooled from two independent experiments and shown as means  ±  SEM. *P*-values were analyzed by one-way ANOVA with Tukey’s post-hoc correction. *P  <  0.05; **P  <  0.01; ***P  <  0.001; ****P  <  0.0001.

To further explore the association between vaccine-induced adaptive immunity and protective effects, we isolated peripheral blood lymphocytes from mice two weeks post-vaccination and two weeks post-H37Ra challenge (28) (Fig. 6A). Cells were stimulated with pool-mix, and T-cell functional profiles were assessed. Compared with T-cell immune responses before infection (Fig. 6B through E), mice immunized with Ad-8^CP^R2 exhibited a further increase in the frequency of CD4^+^ IL-17A^+^ T cells after infection, along with a significant elevation in CD8^+^ IFN-γ^+^ T cells (Fig. 6F through I). Notably, these post-infection frequencies were markedly higher in the Ad-8^CP^R2 group than in all other vaccine groups (Fig. 6F through I).

We also observed that *M. tuberculosis* infection led to the accumulation of myeloid-derived suppressor cells (MDSCs) in the lungs. MDSCs, which derived from aberrant neutrophil differentiation under pathological conditions, can suppress T-cell function and dampen host anti-infective immunity (29, 30). In this study, monocytic MDSCs (M-MDSCs) were defined as Ly6G^-^Ly6C^hi^ and polymorphonuclear MDSCs (PMN-MDSCs) as Ly6G^+^Ly6C^int^ (31). Flow cytometry analysis showed that by 5 weeks post-infection, the proportions of M-MDSCs and PMN-MDSCs in the lungs were significantly expanded compared with the uninfected group (Fig. S3A). Exhausted T cells, defined as PD1^+^Lag3^+^, also increased markedly post-infection (Fig. S3B). To verify whether the three TB vaccine candidates can attenuate T-cell exhaustion and limit the accumulation of immunosuppressive cells, lung lymphocytes were analyzed by flow cytometry. Vaccination significantly decreased the proportions of both MDSC subsets, with multi-antigen vaccines being more effective. Among all groups, the Ad-8^CP^R2 group showed the lowest proportion of PMN-MDSCs (Fig. 6J through L). Concurrently, vaccination alleviated T-cell exhaustion in the lungs compared with unvaccinated infected mice, and Ad-8^CP^R2 was the most effective in reducing exhausted T-cell proportions (Fig. 6M and N).

In summary, following *M. tuberculosis* challenge, Ad-8^CP^R2 promoted stronger activation of CD8^+^IFN-γ^+^ and CD4^+^IL-17a^+^ T cells, driving robust Th1 and Th17 responses that coordinated adaptive immune protection. Furthermore, Ad-8^CP^R2 more effectively reduced the accumulation of immunosuppressive cells and T-cell exhaustion, thereby dynamically regulating pulmonary T-cell responses. These findings indicate that Ad-8^CP^R2 exerts its protective effects through multi-dimensional regulation of adaptive immunity.

## DISCUSSION

In this study, we constructed three ChAd vector-based TB vaccine candidates and evaluated their immunogenicity and protective efficacy against H37Ra challenge. Among these, the multi-antigen vaccine Ad-8^CP^R2 was identified as the optimal candidate. PADRE peptide enhances its CD4^+^ T cell responses and antigen-specific antibody titers. Meanwhile, mechanistic investigations revealed that the C5 peptide component of Ad-8^CP^R2 enhances the autophagic activity of antigen-presenting cells, improves antigen presentation efficiency, and induces a more potent T-cell-dominant adaptive immune response, thereby contributing to multidimensional protection against *M. tuberculosis* infection.

A variety of platforms, including protein subunits and mRNA, are currently under investigation to overcome the limited immunogenicity of earlier TB vaccine candidates. The Peter Andersen team developed the H56 vaccine using a strategy that incorporates antigens expressed during multiple stages of *M. tuberculosis* infection. Compared with H1, which uses only early secretory antigens, H56 activated a higher proportion of CD4^+^ T cells and induced a more robust, multi-dimensional adaptive immune response by engaging polyfunctional T cells, thereby reducing bacterial load in the lungs of infected mice (32). In our construct, we similarly included the early secretory antigen Ag85B and the dormancy-associated protein Rv2628, and further incorporated RpiB, an antigen involved in *M. tuberculosis* metabolism and amino acid synthesis, along with the C5 and PADRE peptides. All three antigens induced strong total IgG and multi-subclass IgG responses. Consistent with the results reported by Haican Liu’s group (33), Ag85B and Rv2628, in either protein or adenovirus-vectored formats, markedly promoted the production of the intracellular cytokines IFN-γ, TNF-α, and IL-17a, indicating Th1 and Th17 responses. In contrast, RpiB protein elicited weaker T-cell responses, primarily stimulating CD8^+^ T cells to mount a Th1-type response and inducing T cells to release IL-17a, thereby eliciting a Th17-type T cell immune response.While many novel TB vaccine candidates focus on CD4^+^ T-cell responses, their protective efficacy remains insufficient to prevent TB infection (34). Our ChAd vector candidate mediated a multi-dimensional T-cell response, not only activating antigen-specific CD4^+^ T cells but also inducing a potent CD8^+^ T-cell response through the differentiation of cytotoxic T lymphocyte (CTL). CD8^+^ T cells contribute to *M. tuberculosis* control by producing IFN-γ, which enhances the antibacterial capacity of macrophages (35). The critical role of CD8^+^ T cells is further underscored by studies from Flynn and colleagues, who demonstrated that antibody-mediated CD8^+^ T-cell depletion in cynomolgus macaques prior to *M. tuberculosis* challenge resulted in larger and more numerous lung granulomas, higher bacterial burdens in the lungs, and increased dissemination to lymph nodes and other organs (36).

The autophagy-enhancing property of the C5 peptide, derived from the *M. tuberculosis* RD1 region antigen CFP10₃₅–₇₅, was previously described by Jagannath and colleagues, who showed that C5 activates the TLR2 pathway and enhances autophagy in dendritic cells *in vitro*, thereby improving antigen presentation efficiency (22). Compared with a single-antigen vaccine, fusion of an antigen with the C5 peptide promoted autophagy, enhanced antigen presentation, strengthened vaccine-specific T-cell responses, and ultimately reduced bacterial load in mice, conferring effective protection against *M. tuberculosis* challenge (23). In the present study, we extended these findings by investigating which antigen-presenting cells are activated by C5 via enhanced autophagy *in vivo* and their specific roles. By designing the multi-antigen candidate Ad-8^CP^R2 incorporating the C5 peptide, we found that immunization induced polarization of macrophages towards the M1 phenotype and promoted the differentiation of dendritic cells into cDC1s within the draining lymph nodes. This significantly enhanced MHCI pathway antigen presentation efficiency and mediated a stronger antigen-specific T-cell response.

TB vaccine development primarily aims to activate antigen-specific Th1 and Th17 immune responses. Th1 responses, mainly mediated by IFN-γ^+^ T cells, limit the spread of *M. tuberculosis* within the lungs and reduce pulmonary inflammation and pathological damage (37). Th17 responses, mediated by IL-17^+^ T cells, modulate mucosal immunity by recruiting innate immune cells to the lungs, contributing to pathogen resistance and maintaining mucosal homeostasis (38). In this study, we analyzed the correlation between lung-infiltrating IFN-γ^+^ and IL-17^+^ TEM cell subsets and the extent of pulmonary inflammation post-infection. The expansion of these TEM subsets showed a negative correlation with inflammatory infiltration, suggesting a protective role mediated through cytokine secretion. Compared with pre-infection levels, the proportions of antigen-specific IFN-γ^+^ CD8^+^ T cells and IL-17^+^ CD4^+^ T cells were significantly increased post-challenge. IFN-γ^+^ CD8^+^ T cells confer immunomodulatory functions by activating the JAK-STAT pathway, enhancing leukocyte migration, and inducing an anti-infective state (39). IL-17 produced by CD4^+^ T cells can act on naive CD4^+^ T cells to facilitate their activation (40), and also enhance the microbicidal functions of neutrophils and macrophages while regulating neutrophil recruitment and mucosal immunity (41). These findings indicate that our vaccine candidate confers protection by activating CTL-mediated bacterial clearance and by producing IL-17 to modulate mucosal immunity, thereby limiting disease progression.

In chronic infections such as hepatitis B virus, human immunodeficiency virus, and COVID-19, neutrophils can aberrantly differentiate into MDSCs under pathological conditions. MDSCs suppress T-cell immunity through various mechanisms (42) and can upregulate PD1 expression on T cells, contributing to T-cell exhaustion (43). In our study, we observed significant accumulation of MDSCs in the lungs of *M. tuberculosis*-infected mice, which was markedly reduced by vaccination. Concurrently, the proportion of exhausted T cells (PD1^+^Lag3^+^) was also decreased, particularly in the Ad-8^CP^R2 group. These findings suggest that the vaccine candidate enhances T-cell immunity while limiting MDSC accumulation and subsequent T-cell exhaustion, thereby contributing to its protective efficacy. However, the precise immunological mechanisms by which T-cell responses reduce MDSC accumulation warrant further investigation.

Notably, although the single-antigen vaccine Ad-8 induced significantly higher Ag85B-specific binding antibody titers than the multi-antigen candidates, the latter demonstrated superior protective efficacy. In the development of antiviral vaccines (e.g., COVID-19, influenza), neutralizing antibodies play a key role by blocking receptor engagement and directly inhibiting viral infectivity (44, 45). Our *in vitro* experiments demonstrated that serum from Ad-8^CP^R2-immunized mice produced antigen-specific antibodies capable of resisting *M. tuberculosis* infection, and antibody levels correlated negatively with the extent of lung inflammation. This suggests that the vaccine candidate induces functionally relevant antibodies that contribute to protection by preventing initial infection and mediating anti-infective immune functions.

Our study has some limitations. First, in the challenge infection experiments, we chose the attenuated strain H37Ra instead of the virulent H37Rv to establish the mouse infection model. In future studies, we plan to employ multiple *M. tuberculosis* strains to comprehensively evaluate the protective efficacy of the candidate vaccines. Second, as previously reported by Clark-Curtiss JE’s group (46), H37Ra can establish long-term colonization in the lungs of mice following intranasal administration, which meets the requirements for constructing a latent *M. tuberculosi*s infection model. However, our current data only demonstrate that the vaccine confers protective efficacy during the acute phase of H37Ra infection, as evidenced by reduced bacterial loads and alleviated pulmonary inflammation and pathology. The actual protective effect of this tuberculosis vaccine candidate in the latent infection model warrants further in-depth investigation in subsequent studies.

In summary, this study demonstrates that a multi-antigen strategy represents a promising approach for TB vaccine design. By comprehensively exploring the key correlates of antigen-specific immune responses that mediate protection in viral-vectored TB vaccines, our work offers new insights for the evaluation and development of next-generation TB vaccine candidates.

## MATERIALS AND METHODS

### Cell lines and bacteria

HEK-293, DC2.4 cells, and Raw264.7 (kindly provided by Dr. Li Long, Tianjin Medical University) were cultured in Dulbecco’s Modified Eagle Medium supplemented with 10% (v/v) fetal bovine serum and 1% antibiotics at 37°C and 5% CO_2_. *M. tuberculosis* H37Ra and H37Ra-eGFP strain were purchased from Gene-Optimal Science & Technology Co., Ltd (Shanghai, China).

### Mouse

Female BALB/c mice (6–8 weeks old) were purchased from GemPharmatech Co. Ltd. (Jiangsu, China) and housed in specific pathogen-free (SPF) animal facilities at Tianjin Medical University, Tianjin, China. Experiments with H37Ra challenge were conducted in the biosafety level 2 animal facilities at the Shanghai Public Health Clinical Center (SPHCC), Fudan University.

### Vaccine construction

Full-length gene sequences of Ag85B, RpiB, and Rv2628 (GeneBank: NC_000962.3) were inserted into the E1 deletion region of ChAd68 vector. The C-terminus of the Ag85B protein was fused with C5 and PADRE peptides via linkers, as illustrated in Fig. 1A. The resulting open reading frame was driven by a cytomegalovirus (CMV) promoter and terminated with a BGH polyadenylation signal. Recombinant adenoviruses were packaged, rescued, and amplified in HEK-293 cells, purified by CsCl gradient ultracentrifugation. The infectious units were titrated on HEK-293 cells using QuickTiter Adenovirus Titer Immunoassay Kit.

### Western blot

HEK-293 and BMDC cells were lysed with RIPA for 20 min after infected with vaccine candidates. Added the 1× loading buffer and heated for 10 min at 95℃. Proteins were separated by SDS-PAGE, transferred to PVDF membranes, and probed with primary antibodies against Ag85B (Abcam, ab43019), Lc3B (CST, 3868), p62 (CST, 23,214), Beclin-1 (CST, 3495), and RpiB, followed by appropriate HRP-conjugated secondary antibodies: anti-rabbit IgG antibody (Abcam, ab6702) or anti-rabbit IgG antibody (Abcam, ab6789). α-Tubulin (Proteintech 66031) and GAPDH (Proteintech 60004) were selected as internal controls.

### Elisa

ELISA plates were coated with Ag85B, RpiB, and Rv2628 (GenScript Biotechnology, Jiangsu, China) proteins at 200 ng/well and incubated overnight at 4°C. Serum samples were serially diluted twofold starting at a 1:100 in PBS containing 1% BSA and added to the plates. After incubation at 37°C for 2 h, secondary antibodies were added: HRP-conjugated goat anti-mouse IgG antibody (Abcam, ab6789) with a 1:100,000 dilution or HRP-conjugated goat anti-mouse IgG1 (Southern Biotech, 1144-05), IgG2a (Southern Biotech, 1155-05), IgG2b (Southern Biotech, 1186-05), IgG3 (Genuin Biotech, 919-HRP) antibody with a 1:5,000 dilution. Plates were further incubated at 37°C for 1 h. After washing, TMB substrate (NCM Biotech) was added, and the reaction was stopped with 2 M H₂SO₄. The optical density (OD) 450–630 was recorded using a microplate reader (Tecan, Switzerland). The endpoint titer was defined as the reciprocal of the highest serum dilution that yielded an absorbance greater or equal to 0.1 OD unit above the control value.

### Prevention of H37Ra infection test

Serum samples were serially twofold diluted and incubated with the H37Ra-eGFP strain (100 CFU/well) for 5 h. After incubation, the mixture was co-cultured with Raw264.7 cells (2 × 10⁵/well) for 24 h. Following five washes with PBS, the infection rate was assessed by fluorescence microscopy. The serum dilution that reduced the infection rate by 50% relative to serum-free controls was determined.

### Immunophenotyping by flow cytometry

All staining procedures were performed at 4°C in the dark. Cell samples were diluted in MACS buffer (PBS with 5% BSA, 50 mM EDTA-Na₂), blocked with anti-CD16/32 antibody (Invitrogen, 14-0161-85), and stained with LIVE/DEAD viability dye (Invitrogen, L2305).

For lymphocytes in PBMCs, surface staining was performed using antibodies against CD3e-PerCP-Cy5.5, CD4-FITC, and CD8-AF700. TEM cells in splenocytes and lung lymphocytes were identified using CD3e-PerCP-Cy5.5, CD4-FITC, CD8-AF700, CD44-PE-eFluor610, and CD62L-APC antibodies. TRM cells were identified using CD3e-PerCP-Cy5.5, CD4-APC-Cy7, CD8-AF700, CD44-FITC, CD62L-APC, CD103-BV605, and CD69-PE-eF610 antibodies. TCM cells were identified using CD3e-PerCP-Cy5.5, CD4-FITC, CD8-AF700, CD44-PE-eFluor610, CD62L-APC, and CCR7-PE-Cy7 antibodies. After staining at 4°C in the dark for 1 h, cells were treated with fixation/permeabilization solution (BD Biosciences, 5125270) and stained for cytokines using TNF-α-BV605, IL-17a-PE, IL-4-BV421 or IL-4-PE-Cy7, IFN-γ-APC or IFN-γ-APC-Cy7. Exhausted T cells were identified using CD3e-PerCP-Cy5.5, CD4-APC-Cy7, CD8-AF700, PD1-PE-Cy7, and Lag3-APC antibodies. MDSCs were identified using CD45-FITC, CD11b-PE-Cy7, Ly6C-PE-eFluor610, and Ly6G-APC antibodies. The gating strategy was shown in Fig. S4.

Lymph node cells were surface-stained using TCR-β-PE, CD19-PerCP-Cy5.5, CD11b-PE-Cy7, CD11c-APC, F4/80-APC-Cy7, CD80-BV510, CD86-BV421, CD206-AF700, CD8a-AF700, and CD63-PE antibodies. The gating strategy was shown in Fig. S5.

Finally, samples were fixed with 2% PFA for 1 h and analyzed using a BD LSRFortessa flow cytometer.

### Immunofluorescent staining

DC2.4 cells were cultured on polylysine-treated coverslips for 1 h at 37°C, 5% CO₂. Cells were then infected with vaccine candidates (MOI = 10:1). After 4 h, cells were fixed with pre-chilled methanol for 5 min, blocked 30 min, and incubated overnight with an anti-LC3B antibody (CST, 3868), then stained with the corresponding secondary antibody for 1 h and observed under a confocal microscope.

### Statistical analysis

All data were presented as mean ± SEM. Statistical significance as shown: *P < 0.05, **P < 0.01, ***P < 0.001, **P < 0.0001. Group comparisons were performed using either unpaired Student’s *t*-test (for two-group analyses) or one-way ANOVA with Tukey correction (for multi-group comparisons), following verification of normality. All statistical analyses were conducted using GraphPad Prism 10 software.

## Data Availability

All data from this study are included within this manuscript/Supplemental Material and are available from the Lead Contact (Dongming Zhou, zhoudongming@tmu.edu.cn) upon request.

## SUPPLEMENTAL MATERIAL

Fig. S1 spectrum.00907-26-s0001.pdf TB vaccine candidates failed to induce central memory T cell immune responses.

Fig. S2 spectrum.00907-26-s0002.pdf Vaccine candidates tend to enhance antigen presentation efficiency by activating the MHCI antigen presentation pathway rather than MHCII.

Fig. S3 spectrum.00907-26-s0003.pdf *M. tuberculosis* infection led to the accumulation of myeloid-derived suppressor cells and increased proportion of exhausted T cells.

Fig. S4 spectrum.00907-26-s0004.pdf Gating strategy for measuring memory T cells.

Fig. S5 spectrum.00907-26-s0005.pdf Gating strategy for measuring macrophages and dendritic cells.

ASM does not own the copyrights to Supplemental Material that may be linked to, or accessed through, an article. The authors have granted ASM a non-exclusive, world-wide license to publish the Supplemental Material files. Please contact the corresponding author directly for reuse.
