# Supplementary material for: An adenovirus-based multiantigen vaccine protects against tuberculosis by coordinating adaptive immunity and curbing immunosuppressive mechanisms
Source: Microbiol Spectr. 2026 Aug 28;14(10):e00907-26. doi: 10.1128/spectrum.00907-26 (PMC13688876; doi:10.1128/spectrum.00907-26)
Supplement: Supplementary file 1 [file spectrum.00907-26-s0001.pdf]

Supplementary information

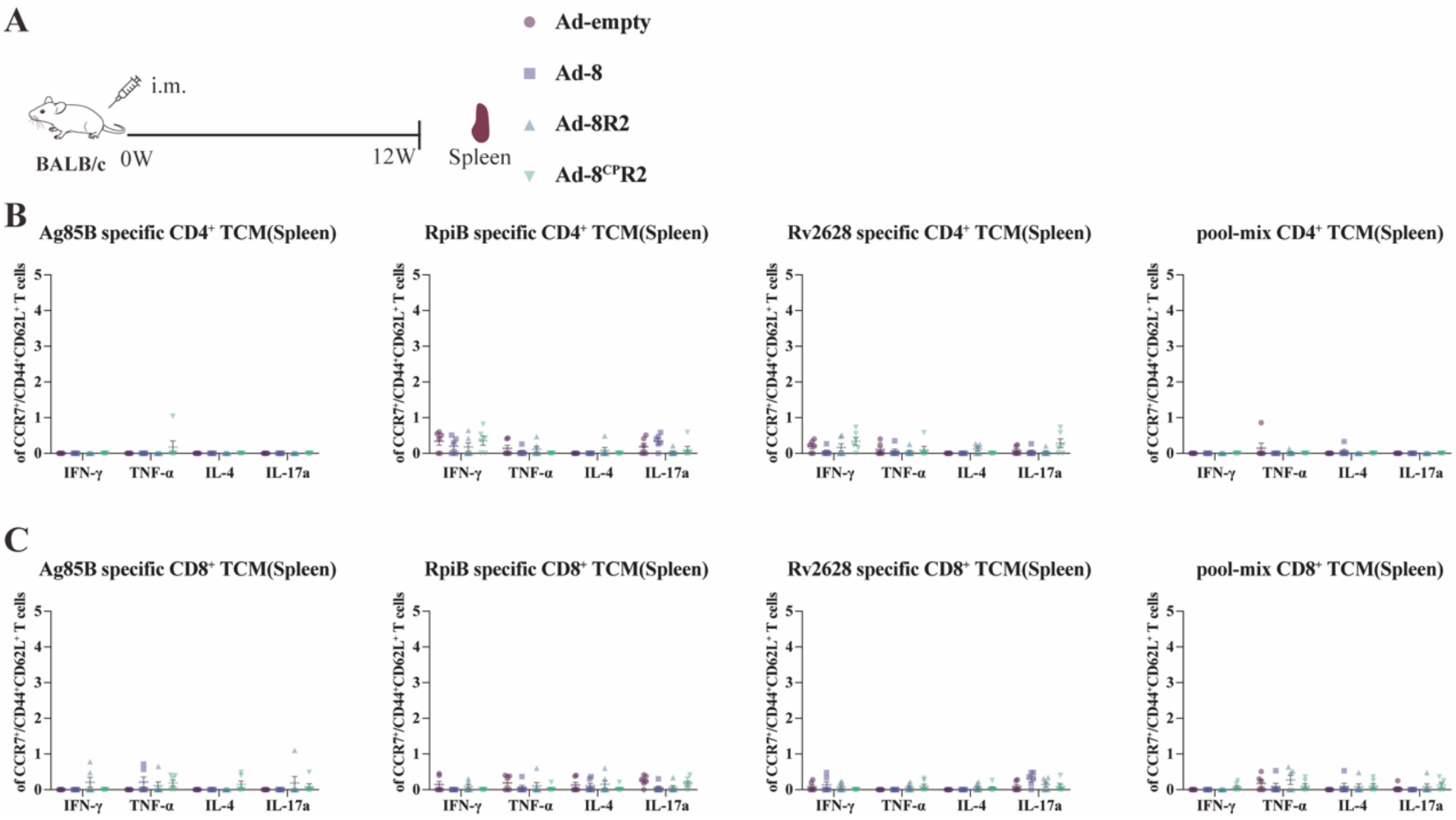

Fig. S1. TB vaccine candidates failed to induce central memory T cells immune responses

(**A**) Immunization schedule. Mice were intramuscularly (i.m.) injection immunized with $1*10^8$ IFU of each vaccine candidate. Ad-empty was used as control ($1*10^8$ IFU). (**B and C**) The frequencies of cytokine-producing, antigen-specific $CD4^+$ (**B**) and $CD8^+$ (**C**) TCM cells. Splenocytes were isolated form BALB/c (*n*=6) at 12 weeks post-immunization and stimulated with peptide pools specific for Ag85B, RpiB, Rv2628, or pool-mix. All data were shown as means ± SEM. *P*-values were analyzed by one-way ANOVA with Tukey correction.
