# Supplementary material for: An adenovirus-based multiantigen vaccine protects against tuberculosis by coordinating adaptive immunity and curbing immunosuppressive mechanisms
Source: Microbiol Spectr. 2026 Aug 28;14(10):e00907-26. doi: 10.1128/spectrum.00907-26 (PMC13688876; doi:10.1128/spectrum.00907-26)
Supplement: Supplementary file 2 [file spectrum.00907-26-s0002.pdf]

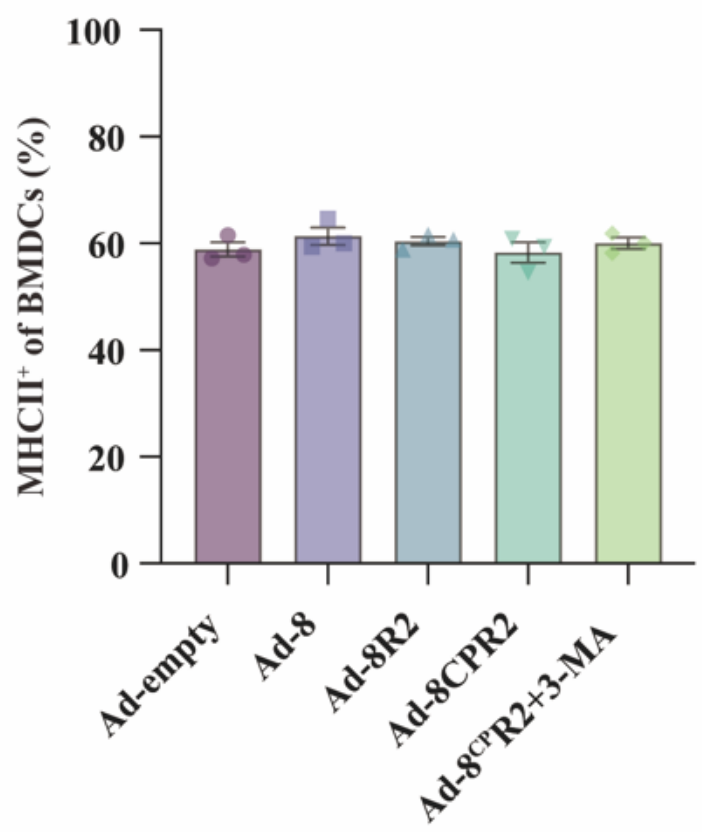

Fig. S2. Vaccine candidates tend to enhance antigen presentation efficiency by activating the MHCI antigen presentation pathway rather than MHCII

Quantifation of the proportion of MHCII$^+$ BMDCs. All data were shown as means ± SEM. *P*-values were analyzed by one-way ANOVA with Tukey correction.
