# Supplementary material for: An adenovirus-based multiantigen vaccine protects against tuberculosis by coordinating adaptive immunity and curbing immunosuppressive mechanisms
Source: Microbiol Spectr. 2026 Aug 28;14(10):e00907-26. doi: 10.1128/spectrum.00907-26 (PMC13688876; doi:10.1128/spectrum.00907-26)
Supplement: Supplementary file 3 [file spectrum.00907-26-s0003.pdf]

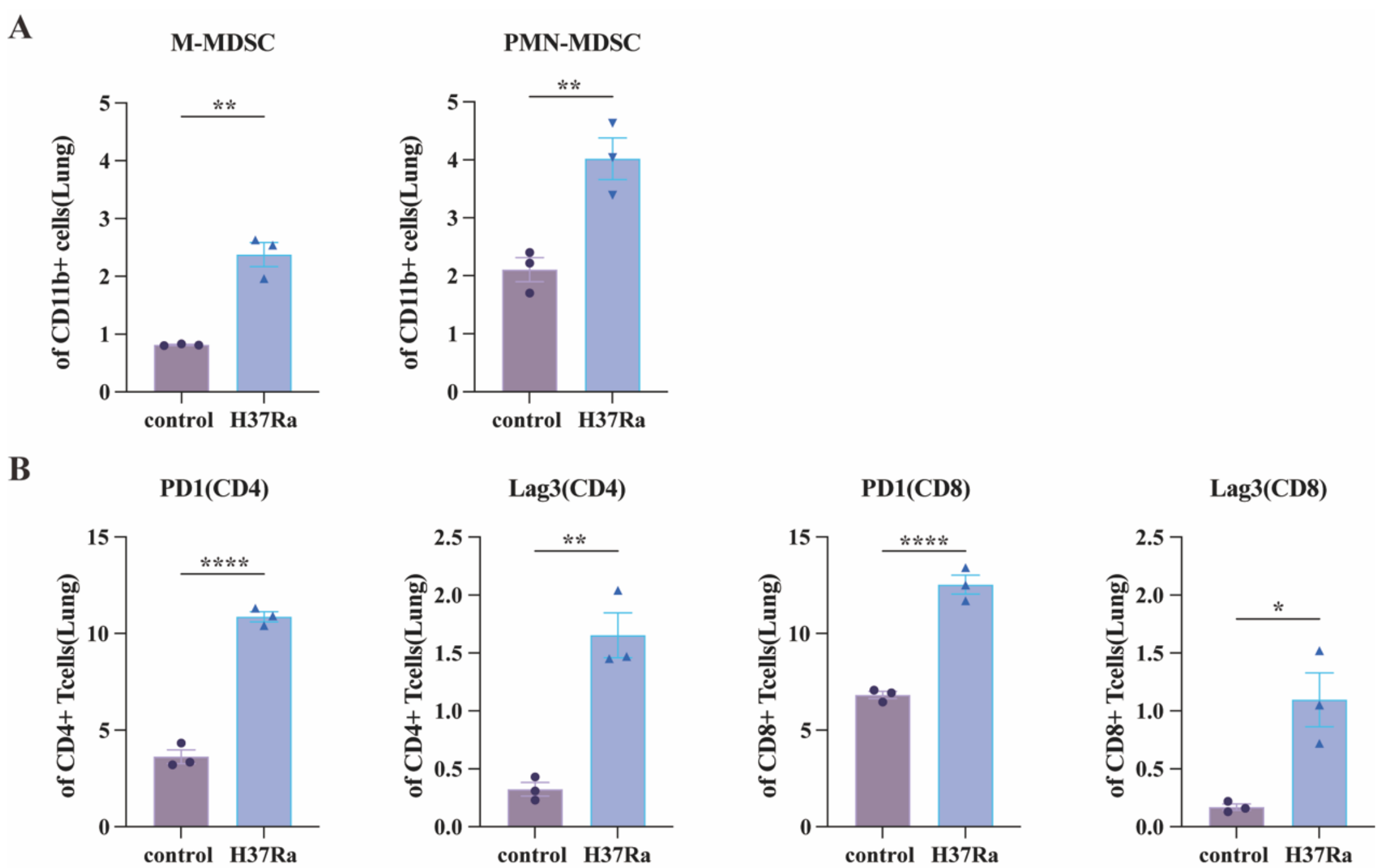

Fig. S3. ***M. tuberculosis*** **infection led to the accumulation of myeloid-derived suppressor cells and increase proportion of exhausted T cells**

(**A**)The proportion of M-MDSCs and PMN-MDSCs in the lungs of mice (*n*=3). (**B**) The frequencies of exhausted $CD4^{+}$ T cells and $CD8^{+}$ T cells in lungs from BALB/c mice (*n*=3). Statistical analysis was conducted using unpaired Student's t-test (for two-group analyses) and represented as mean ± SEM. $*P \leq 0.05$, $**P \leq 0.01$, $***P \leq 0.001$, and $****P \leq 0.0001$.
