# Supplementary material for: An adenovirus-based multiantigen vaccine protects against tuberculosis by coordinating adaptive immunity and curbing immunosuppressive mechanisms
Source: Microbiol Spectr. 2026 Aug 28;14(10):e00907-26. doi: 10.1128/spectrum.00907-26 (PMC13688876; doi:10.1128/spectrum.00907-26)
Supplement: Supplementary file 4 [file spectrum.00907-26-s0004.pdf]

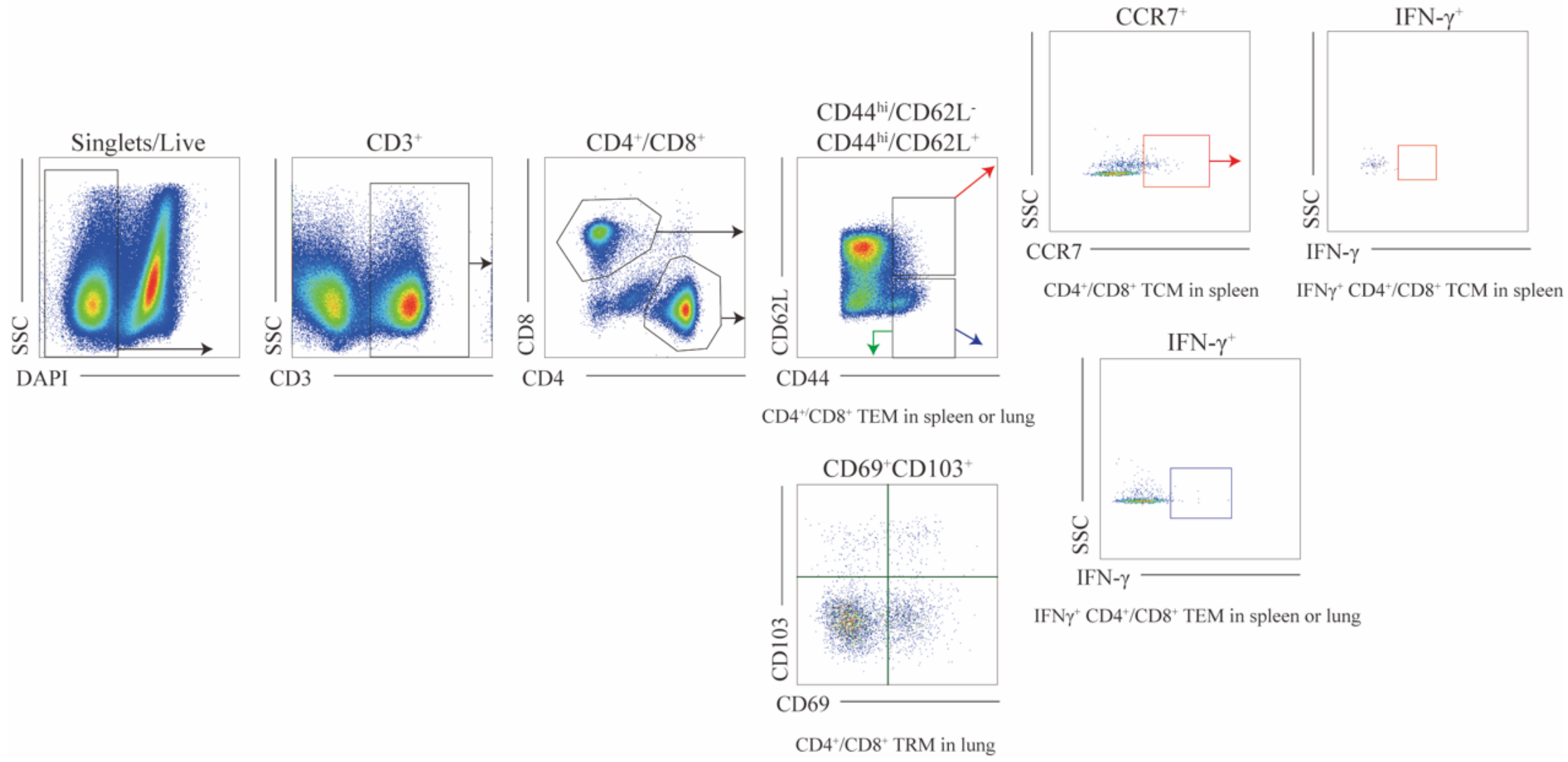

Fig. S4: Gating strategy for measuring memory T cells

Representative contour plots of splenic $CD8^+$ TCM cells ($CD8^+$ $CD44^{hi}$ $CD62L^+$ $CCR7^+$) and $CD4^+$ TCM cells ($CD4^+$ $CD44^{hi}$ $CD62L^+$ $CCR7^+$); splenic and pulmonary $CD8^+$ TEM cells ($CD8^+$ $CD44^{hi}$ $CD62L^-$) and $CD4^+$ TEM cells ($CD4^+$ $CD44^{hi}$ $CD62L^-$); pulmonary $CD8^+$ TRM cells ($CD8^+$ $CD44^{hi}$ $CD62L^-$ $CD69^+$ $CD103^+$) and $CD4^+$ TRM cells($CD4^+$ $CD44^{hi}$ $CD62L^-$ $CD69^+$ $CD103^+$). The corresponding cytokine-secreted memory T cell populations were gated downstream, respectively.
