# Supplementary material for: An adenovirus-based multiantigen vaccine protects against tuberculosis by coordinating adaptive immunity and curbing immunosuppressive mechanisms
Source: Microbiol Spectr. 2026 Aug 28;14(10):e00907-26. doi: 10.1128/spectrum.00907-26 (PMC13688876; doi:10.1128/spectrum.00907-26)
Supplement: Supplementary file 5 [file spectrum.00907-26-s0005.pdf]

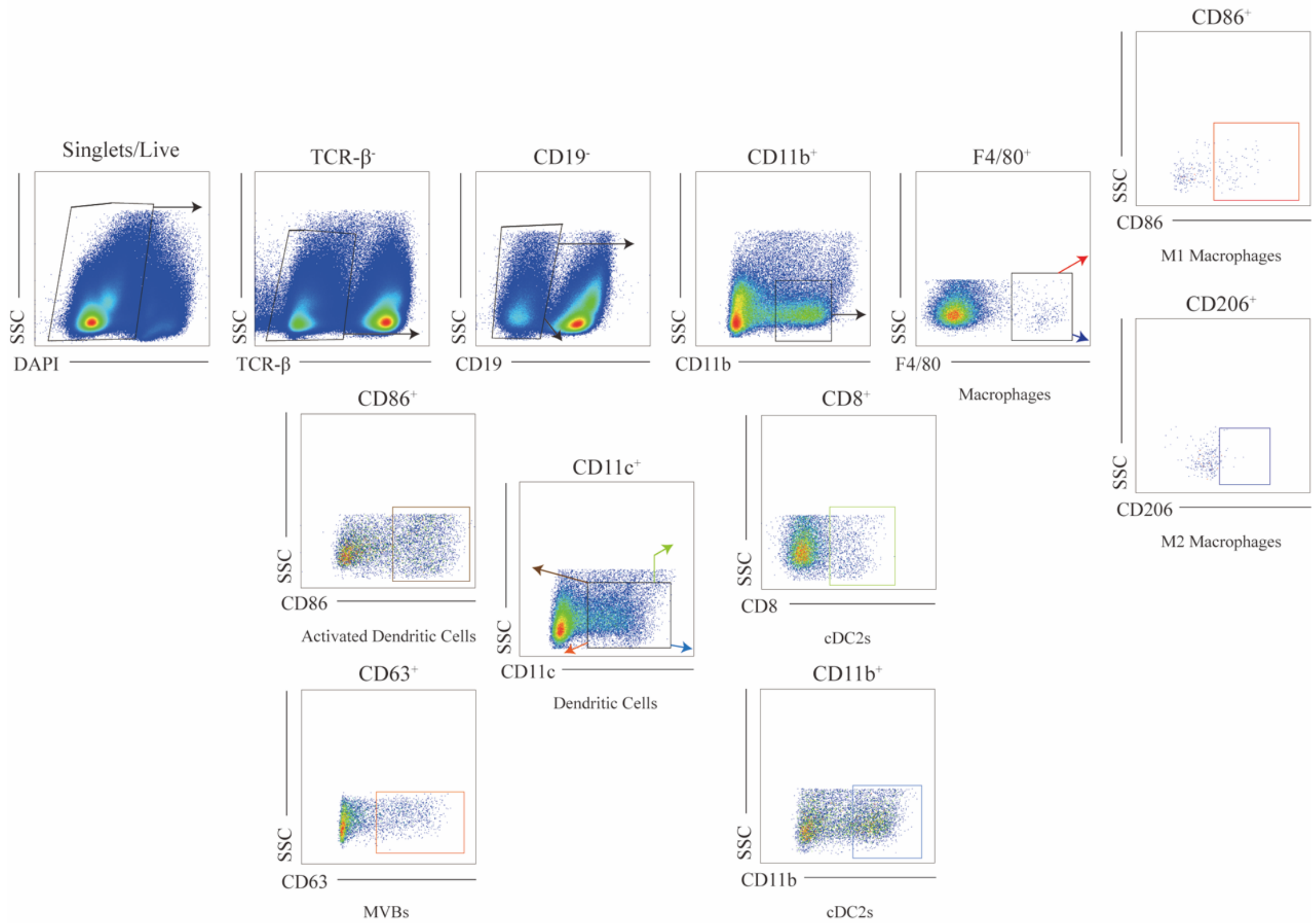

Fig. S5: Gating strategy for measuring macrophages and dendritic cells

Representative contour plots showing the gating strategy for immune cells isolated from draining lymph nodes. Macrophage subsets were identified as M1 ($CD11b^+$ $F4/80^+$ $CD86^+$) and M2 ($CD11b^+$ $F4/80^+$ $CD206^+$). Dendritic cell (DC) subsets were characterized as activated DCs ($CD11c^+$ $CD86^+$), conventional type 1 DCs (cDC1s; $CD11c^+$ $CD8^+$), conventional type 2 DCs (cDC2s; $CD11c^+$ $CD11b^+$), and multivesicular bodies (MVBs) positive cells ($CD11c^+$ $CD63^+$).
